# Editorial: Similarities and Discrepancies Across Family Members at Multiple Levels: Insights From Behavior, Psychophysiology, and Neuroimaging

**DOI:** 10.3389/fpsyg.2021.831048

**Published:** 2022-01-28

**Authors:** Christy Rae Rogers, Yang Qu, Tae-Ho Lee, Siwei Liu, Sun Hyung Kim

**Affiliations:** ^1^Department of Human Development and Family Sciences, Texas Tech University, Lubbock, TX, United States; ^2^School of Education and Social Policy, Northwestern University, Evanston, IL, United States; ^3^Department of Psychology, Virginia Tech, Blacksburg, VA, United States; ^4^Department of Human Ecology, University of California, Davis, Davis, CA, United States; ^5^Department of Psychiatry, University of North Carolina at Chapel Hill, Chapel Hill, NC, United States

**Keywords:** family similarity, parent-child dynamics, parenting, psychological processes, neurobiological processes, positive youth development, quantitative similarity

Family members are responsive to one another not just in what they verbally communicate with one another, but can also be connected through psychological, behavioral, physiological, and neural processes. These complex family dynamics can be represented as similarities and discrepancies between family members in various developmental processes. Such similarities may prepare developing youth to adapt to their family environments, as well as outside environments including schools, neighborhood, and community spaces. As a growing number of studies are examining the role of parent-child concordance or synchrony in youth development (e.g., Lee et al., 2017, 2018; Nguyen et al., 2021), it is critical to identify similarities and discrepancies across family members at different levels (e.g., perception, behavior, biology) to inform our understanding of how families affect adolescent functioning and well-being. There is a need to gather convergent evidence on interpersonal family similarity using a variety of approaches (e.g., observation, survey, psychophysiology, and neuroimaging) across family subsystems. This Research Topic aims to provide as interdisciplinary understanding of how multi-level interpersonal similarity across family members can contribute to youth development.

Novel approaches to examining similarity between family members across various developmental processes and cultural contexts were showcased. Chen and Qu proposed a computational cultural neuroscience approach to better assess family member similarities using sophisticated techniques including representational similarity analysis, real-time synchrony, and hyperscanning. This instrumental review provides a toolkit to better understand similarity between family members through neural and psychological processes, as well as differences across cultural contexts. Additionally, Tagliabue et al. showcased a latent congruence model to estimate parent-child similarity and accuracy in perception of support exchanges between mothers and fathers, and between Italian and German families. This cross-national cross-informant measurement invariance technique can help researchers rigorously compute and evaluate similarities and discrepancies in family dyads across groups based on culture, age, gender, and other sociocultural factors.

The greatest trend in family similarity research includes processes within the parent-child dyad, as reflected in this Research Topic. Ha et al. found that mothers and their children were similar psychologically as evidenced by the importance of taste attributes in decision-making about food choices, as well as biologically as shown by their body mass index. Their contribution highlights the importance of better understanding psychobiological similarities in mother-child dyads as these findings inform the salience of nutrition education for parents to communicate with their children. Alternatively, Zhou et al. investigated similarity, or the lack thereof, in family member perceptions of parental socialization goals of their adolescent children. Discrepancies between mothers and their children in their perceptions of parent socialization goals associated with adolescent mental health problems, such that only adolescent perceptions were indicative of their depressive symptoms. These findings underscore the necessity to understanding the intricacies of how parent-child similarity, and dissimilarity, in developmental processes may affect youth well-being.

Sibling relationships serve as another fruitful context to examine how similarity in developmental processes can affect positive youth development (for a review, Kramer et al., 2019). Shi and Campione-Barr found that sibling temperament similarity and parenting similarity together predicted more positive family dynamics across time, whereas discrepancies in sibling temperament and parenting together also predicted more positive family dynamics across time. This contribution emphasizes the potential benefits of family congruence in similarity and discrepancy, corroborating theoretical frameworks that posit that parenting should be tailored based on the characteristics and needs of each child for optimal development (Chess and Thomas, 1991). Other recent sibling research has utilized representational similarity analysis, as outlined by Chen and Qu, and found that adolescents become more similar to one another in behavioral and neural decision-making processes after watching their older sibling take risks (Rogers et al., 2021). These great strides to better understand similarity between siblings provides a rich perspective on dynamic contributions toward youth development.

Together, these papers emphasize the dynamic and complex nature of how family members affect one another's psychological, behavioral, and neurobiological processing, and that capturing organic family similarities or incongruence informs our understanding of how families influence youth development. Family systems theory highlights that no one individual can be completely understood without taking into account the family system, that the whole family is greater than the sum of all its members, due to the complex relationship history and changing social roles of the family (Cox and Paley, 1997). Furthermore, family subsystems continuously interact bidirectionally within the larger family system, adapting to changes within and outside of the family. Importantly, chronic family discrepancies in psychological, behavioral, and neurobiological processes can cascade to large consequences, particularly for youth. Thus, there is an opportunity to transform family systems theory to encompass the complex developmental processes and sociocultural factors embedded in family dynamics.

We propose the family systems similarity model, an interdisciplinary framework for conceptualizing how developmental processes and sociocultural factors interact with family dynamics to shape the development of youth (Figure 1). Consistent with family systems theory, the family system is represented by bidirectional influences between family members; however, the family systems similarity model illustrates potential similarity in each family subsystem. Furthermore, the family systems similarity model shows that the developmental system, which can be represented as cognitive, psychological, behavioral, physiological, and neural processes, may underlie the mechanism of similarity between family members. The sociocultural system encompasses the socially constructed factors that can differentiate how these similarity processes manifest between family members. The concept of intersectionality is highlighted to convey the unique experiences of each family member, as exemplified by gender, age, ethnicity, and country of origin and inhabitance, amongst a myriad of other possible identity intersections. Together, these three systems continuously fluctuate and interact to shape the development of youth.

**Figure 1:**
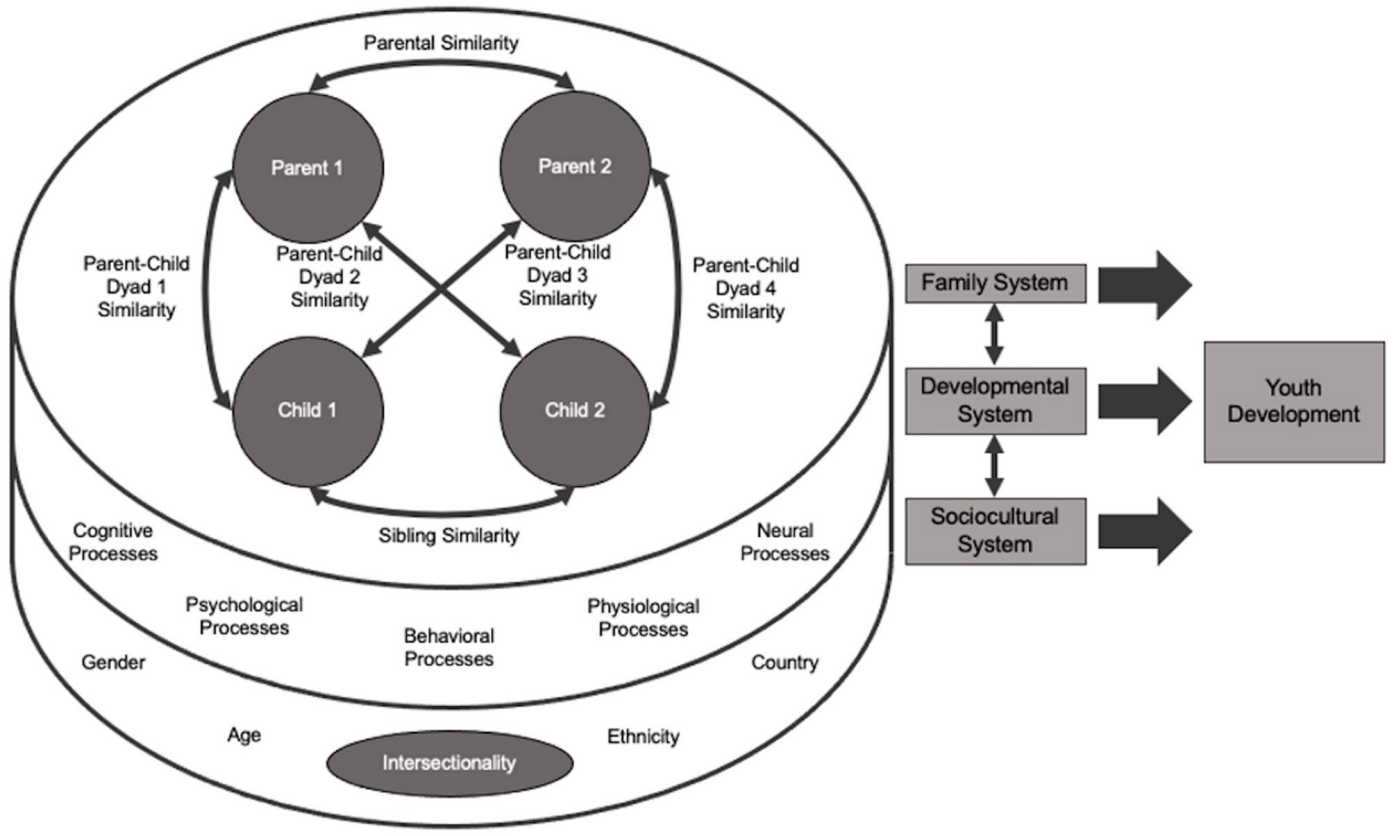
Family systems similarity model.

Of note, the family, developmental, and sociocultural systems are parsimoniously depicted in the family systems similarity model, and as such, we encourage researchers to adapt this model to meet the needs for diversity in families and human development (Parke, 2004). Although the family system illustrates a family of four, this model can also represent families with single-parents, additional caregivers, and any number of children, as well as encompass family members irrespective of biological relatedness and gender. This theoretical model can guide researchers to better understand existing and future patterns of interpersonal family processes, as well as inform strategies for family interventions and therapy as they face stressful life events.

## Author Contributions

YQ and T-HL proposed the scope and concept of the Research Topic, with feedback from CR, SL, and SK. YQ handled submissions. YQ, CR, T-HL, and SL served as editors for submitted manuscripts. CR prepared the main draft of this Editorial, which was reviewed by YQ, T-HL, SL, and SK. All authors contributed to the article and approved it for publication.

## Conflict of Interest

The authors declare that the research was conducted in the absence of any commercial or financial relationships that could be construed as a potential conflict of interest.

## Publisher's Note

All claims expressed in this article are solely those of the authors and do not necessarily represent those of their affiliated organizations, or those of the publisher, the editors and the reviewers. Any product that may be evaluated in this article, or claim that may be made by its manufacturer, is not guaranteed or endorsed by the publisher.
